# Effects of perceptual and decisional uncertainty on serial dependence in orientation perception

**DOI:** 10.3758/s13414-025-03034-5

**Published:** 2025-03-03

**Authors:** Zoë Little, Colin W. G. Clifford

**Affiliations:** 1 School of Psychology, University of New South Wales, Sydney, NSW Australia; 2 UNSW Sydney, F23 Library Walk, Mathews Building, Kensington, NSW 2033 Australia

**Keywords:** Serial dependence, Orientation perception, Sensory uncertainty

## Abstract

Where assimilative serial dependence occurs in perception, responses to visual attributes such as orientation are typically biased towards the attributes of visual stimuli seen in the recent past. Serial dependence is thought to increase the signal-to-noise ratio of visual representations and is reliably increased by uncertainty in the target stimulus. However, the amount of uncertainty in the preceding inducer stimulus does not seem to modulate serial dependence. Here, we consider whether uncertainty in the decision made about the inducer stimulus modulates serial dependence instead by using stimuli with high positive evidence (high-contrast signal plus high-contrast noise) and low positive evidence (low-contrast signal and low-contrast noise). Such stimuli are matched in their signal-to-noise ratio but differ in overall contrast, and have been found to modulate response confidence but not response accuracy. Thirty-three participants viewed oriented Gabor patches presented under different levels of signal-to-noise ratio and positive evidence and reported their orientation, as well as their response confidence. We found that serial dependence was greatest for target stimuli that had low signal-to-noise ratio, low positive evidence, or were responded to with low subjective confidence. We find no modulation of serial dependence by uncertainty of any kind in the inducer trial. This finding suggests that serial dependence may not reflect a bias towards previous decisions, and has implications for wider theoretical accounts of serial dependence. The data and materials for this experiment are available via the Open Science Framework at https://osf.io/kvy4c/

**Supplementary Information:**

The online version contains supplementary material available at 10.3758/s13414-025-03034-5.

## Introduction

We experience the visual world as stable from moment to moment, despite small changes in lighting or viewpoint dramatically changing the retinal image yielded by objects in our environment. That the visual system might exploit the presumed stability of the sensory environment across time to smooth our subjective visual experience is referred to as *assimilative* or *positive serial dependence* (herein: serial dependence). Where serial dependence occurs, responses to visual stimuli are biased towards the attributes of visual stimuli seen in the recent past (Cicchini et al., 2024; Manassi et al., 2023; Pascucci et al., 2023).

This effect is most commonly demonstrated with orientation – when participants are asked to adjust a bar to match the orientation of a briefly presented stimulus (the target), responses are skewed towards the orientation presented on the previous trial (the inducer; e.g., Ceylan et al., 2021; Cicchini et al., 2017, 2018; Fischer & Whitney, 2014; Fritsche & de Lange, 2019). This temporal averaging occurs for similar stimuli presented within a short time window and at similar locations. The combination of the temporal, spatial, and feature similarity ranges within which serial dependence occurs is referred to as the “continuity field” (Fischer & Whitney, 2014; Manassi & Whitney, 2024). The limits of this field are thought to delineate small changes to visual representations that are more likely to be ‘noise’ (i.e., small changes to lighting, or random noise in the visual system) from larger changes to visual representations that are more likely to represent a change in the world. That serial dependence occurs only within this continuity field suggests its functional role in increasing the signal-to-noise ratio (SNR) of visual representations (Cicchini et al., 2017, 2018).

When target stimuli are less precise – for example, a low-spatial frequency or noisy Gabor – the skew towards inducer stimuli is greater (Ceylan et al., 2021; Cicchini et al., 2018; Fulvio et al., 2023; Gallagher & Benton, 2022). This modulation of serial dependence by uncertainty is often taken as further evidence of the functional role of serial dependence in improving perceptual efficiency (Cicchini et al., 2018; Gallagher & Benton, 2022; van Bergen & Jehee, 2019). Under this account, the uncertainty of the inducer stimulus should also matter – averaging a noiseless visual input with a noisy previous visual input would serve to amplify rather than dampen noise. Yet the uncertainty of the inducer stimulus does not seem to modulate the serial dependence effect so consistently: many studies find no effect of uncertainty in the inducer stimulus (Cicchini et al., 2018; Clifford et al., 2018; Gallagher & Benton, 2022).

Whereas the stimulus uncertainty associated with the inducer does not have a clear effect, there could be an effect of uncertainty in the decision made about the inducer. Providing participants with feedback about their accuracy can increase serial dependence (Fornaciai & Park, 2022), and serial dependence may also be greater when one’s subjective confidence in the previous decision is higher (Bosch et al., 2020; Samaha et al., 2019; Suárez-Pinilla et al., 2018). Samaha et al. (2019) examine the role of decision confidence in serial dependence using the positive evidence bias – the finding that accuracy is determined by the balance of evidence for each alternative, while confidence is determined by the absolute magnitude of evidence in favour of a perceptual decision (Koizumi et al., 2015; Maniscalco et al., 2016; Odegaard et al., 2018; Peters et al., 2017; Rausch et al., 2017; Samaha et al., 2016; Samaha et al., 2019; Zylberberg et al., 2012). Increasing the contrast of both the signal and the noise of a stimulus contrast to the same extent, leaving the balance of positive and negative evidence unchanged, has no effect on accuracy but increases subjective confidence. That accuracy is not affected suggests that the quality of the percept is unchanged despite the changes in confidence. Responses made to such “high positive evidence” inducer stimuli exerted a greater attractive effect on subsequent responses than “low positive evidence” inducer stimuli, suggesting that serial dependence is greater following more confident decisions independently of perceptual uncertainty (Samaha et al., 2019).

Here, we test whether serial dependence is modulated by decisional and perceptual uncertainty at different time points – namely, whether uncertain sensory input induces a reliance on more certain previous decisions. This would align with recent models suggesting that serial dependence reflects the reweighting of sensory input by previous decisional traces (Pascucci et al., 2019) and the general idea that the serial dependencies may originate from decision making. We use positive evidence (i.e., contrast) to manipulate decisional uncertainty and SNR to manipulate stimulus uncertainty. We predict that the serial dependence effect should be increased by high positive evidence in the inducer trial as well as low SNR on the target trial.

## Method

### Participants

Thirty-three participants (five male, 25 female, three other, *M*_*age*_ = 18.9 years, *SD*_*age*_ = 1.7 years) completed the study. Participants were undergraduate psychology students from the University of New South Wales (UNSW) who received course credit for their participation. This was the sample after three participants were excluded: two because their mean adjustment errors were greater than 2 standard deviations over the group mean error, and one for mainly using the ‘4’ confidence response (on 91.4% of trials). All participants gave informed consent, and the study was approved by the UNSW ethics committee (HREAP C: Behavioural Sciences, reference 5743).

### Materials

Stimuli were presented on a linearized Cambridge Research Systems Display +  + LCD 32-in. computer monitor with resolution 1,920 × 1,080 (28 pixels/cm, refresh rate 120 Hz). Participants sat in a chin rest to ensure they were 57 cm away from the screen. Stimuli were Gabor patches with a spatial frequency of 0.5 cycles per degree presented at 8° × 8° of visual angle and at 6.5° of visual angle to the left or right of the centre of the screen (counterbalanced between blocks). Gabor stimuli were presented on a uniform grey background at their mean luminance (60 cd/m^2^). The task was programmed in MATLAB 2024a using Psychtoolbox (Brainard, 1997; Kleiner et al., 2007).

**Fig. 1:**
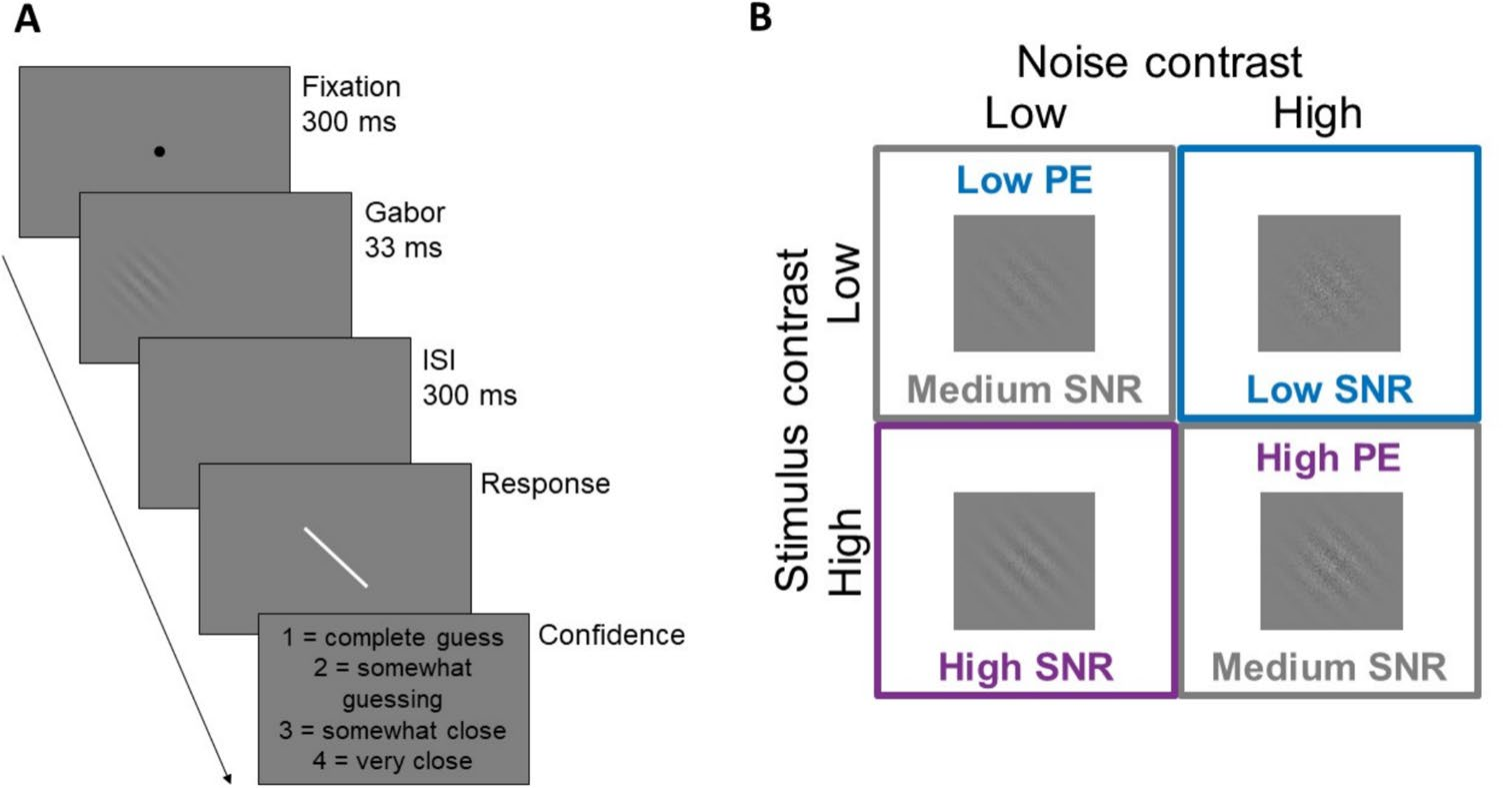
Trial structure and manipulations of signal-to-noise ratio (SNR) and positive evidence (PE). **A**. The structure of a single trial in the task. All stimuli (fixation, Gabor, response bar, and confidence prompts) have been represented on a larger scale than they were actually presented for the sake of visibility. **B**. Examples of all possible combinations of stimulus and noise contrast and the SNR and PE conditions they give rise to

The contrast and SNR of the stimuli were manipulated. The stimuli were presented at one of two levels of contrast (prior to averaging with the noise masks): 8.5% (high contrast, 50% of trials) or 4.25% (low contrast). These are the average contrast values used by Samaha et al. (2019), yielded through a staircasing procedure. Stimuli were also presented under one of two levels of noise (prior to averaging with the Gabor): either overlaid with a Gaussian mask presented at 100% contrast (high noise, 50% of trials) or 50% contrast (low noise). The noise and contrast on each trial was chosen at random. The combination of the contrast and noise of a stimulus determined the trial’s SNR and positive evidence as illustrated in Fig. 1. There were three possible levels of SNR on each trial (low, medium, and high) and the positive evidence was taken as the overall contrast when the SNR was medium: a medium SNR stimulus could be presented with overall high contrast (high positive evidence) or low contrast (low positive evidence). The level of SNR and/or positive evidence on any given trial was random.

### Procedure

A single trial began with the presentation of a fixation point for 300 ms, followed by the Gabor stimulus for 33 ms. Following a 300-ms interstimulus interval (ISI), participants reported the orientation of the Gabor stimulus by adjusting a response bar. This bar was the same length as the diameter of the Gabor stimuli and was 1° wide. The orientation at which this bar began on each trial was selected randomly between 0° and 179°. Participants adjusted the bar by moving the mouse then clicked once to record their response. Immediately following this, participants reported their confidence in their response using the keys 1 (complete guess), 2 (somewhat guessing), 3 (somewhat close), or 4 (very close). Participants were reminded on each trial the label associated with each confidence response.

The orientation of the stimulus on the first trial was 90°, and the orientation on all following trials was pseudorandomly chosen to be within ± 30° of the previous stimulus in intervals of 5°. Each of the 13 possible differences in orientation between trials was shown 40 times for a total of 520 trials. Participants completed 15 practice trials before the task began. Participants had the opportunity to take a self-paced break after every 52 trials. The task took participants an average of 38.1 min (*SD* = 7.7 min) to complete.

### Data analysis

#### Positive evidence bias analysis

Following Samaha et al. (2019), we used several metrics to check that confidence differed between the two levels of positive evidence but accuracy did not. We looked at average confidence and error (median and mean) on low and high positive evidence trials, as well as the precision and guess rate in each condition obtained from a mixture model fit to the distribution of errors made by each participant (Bays et al., 2009). This model fits a mixture of a von Mises and a uniform distribution to the angular errors in responses made using an expectation-minimisation algorithm. The MATLAB code for this model was sourced from www.bayslab.com. Log-transformed values of guess rate were used in all analyses. We used Bayes factors to examine the evidence for the absence of the effects in these analyses. Bayesian analyses were carried out in RStudio 2024.04.2 using the *BayesFactor* package (or the Bayesian rank-based Spearman’s rho procedure provided by van Doorn et al. (2020) for the correlational analyses). We interpret BF_10_ < 0.33 as evidence for the null hypothesis, and BF_10_ > 1 as evidence for the alternative hypothesis.

#### Serial dependence data processing

All serial dependence analyses were conducted in MATLAB 2024a. Analysis scripts and data can be found at https://osf.io/kvy4c/. To account for any overall clockwise or counter-clockwise biases shown by participants, we calculated for each participant the circular mean of their errors and subtracted this from their raw responses. The first trial of each block was not included in the final analysis of serial dependence as it had no “inducer trial”. We removed trials on which the response time was faster than 200 ms. Following previous literature, we removed trials with extreme errors (e.g., Fritsche et al., 2017; Pascucci et al., 2019; Samaha et al., 2019). Within each condition of interest (i.e., each level of SNR and positive evidence), we calculated each participant’s mean error and removed trials from the analysis where the angular error was greater than two standard deviations above the participant’s mean for that condition, assuming these large errors reflect lapses in participants’ attention. On average, these criteria removed 4.2% of trials. Supplementary Analysis 5 (see Online Supplementary Material) shows that most reported effects were still present when all valid trials were analysed.

To measure serial dependence to previous stimuli, we calculated the difference in orientation between the target and inducer stimulus on each trial by subtracting the former from the latter. A positive difference indicates that the inducer stimulus was more clockwise than the target stimulus, while a negative difference indicates that the inducer stimulus was less clockwise than the target stimulus. We also calculated each participant’s response error on each trial (the angular distance between where the adjustment bar was placed and the orientation at which the stimulus was presented) by subtracting the orientation of the stimulus from the response made, such that a positive difference indicates that the bar was placed more clockwise that the stimulus was.

We were also interested in measuring serial dependence effects to the response, rather than the stimulus, on the inducer trial. The orientation of the inducer response may be a better representation of the percept experienced by the participant than the inducer stimulus is, and response-based analyses have been shown to yield larger effects than stimulus-based analyses (Fründ et al., 2014; Gallagher & Benton, 2022, 2024; Moon & Kwon, 2022; Pascucci et al., 2019; Sadil et al., 2024). Therefore, we also calculated the difference between the target trial’s stimulus orientation and the previous trial’s response, rounding the difference values to the nearest multiple of 5 and only fitting the data to differences within the range − 30° to 30° to match the stimulus-based analysis. Response-based analyses are subject to spurious effects that resemble serial dependence due to idiosyncratic response biases in reporting orientation that do not actually depend on trial order (Fritsche, 2016; Gallagher & Benton, 2022, 2024; Pascucci et al., 2019; as demonstrated in Supplementary Analysis 2 (Online Supplementary Material)). To remove these spurious effects, we fit a sum of three sinusoids to the angular errors made at each orientation for each participant, then removed the errors predicted by this model from the actual response made on each trial (as in Gallagher & Benton, 2022, 2024; Pascucci et al., 2019). As these responses biases are likely to change under differing levels of uncertainty (Tomassini et al., 2010), we did this separately for each possible level of uncertainty on the target trial (low SNR, medium SNR with low-contrast (low positive evidence), medium SNR with high-contrast (high positive evidence), and high SNR). Supplementary Analysis 2 (Online Supplementary Material) shows that spurious significant effects that resemble serial dependence were successfully removed by residualizing the data like so, as there were no longer significant effects found in shuffled data where the inducer trial did not actually precede the target trial in practice. We then analysed the residualised data using the methods described in the next section.

#### Serial dependence analysis

We analysed the serial dependence effect in several ways. First, we fit the relationship between angular error and orientation difference with a derivative of von Mises function, the circular analogue of a normal distribution, wherein orientation wrapped around 180°. The amplitude of this function is taken as the strength of the serial dependence bias, and the location on the x-axis where this maximum amplitude occurs is taken as the stimulus difference at which the serial dependence effect is maximal. A third parameter reflected the vertical offset of the data and accounted for any further clockwise or counterclockwise biases. We fit the data to the aggregate of all participants’ data, and smoothed the data with a moving average (bin size = 5) to improve the variance accounted for by the model.

Next, to replicate the analyses of Samaha et al. (2019) and others, we fit the data with a first derivative of Gaussian model. Again, this model had three parameters we allowed to vary, corresponding to the amplitude of the function (the magnitude of the serial dependence), the sigma (from which we derive the location on the x-axis at which the function peaks), and a vertical offset. As for the derivative of von Mises model, we smoothed the data with a moving average (bin size = 5) to improve the variance explained. As this model is not constrained to wrap around the orientations sampled as the derivative of von Mises is, the bootstrapping procedure described in the *Statistical analysis* section used to create confidence intervals (CIs) of the effect often yielded very large amplitude values. For this reason, we consider the derivative of von Mises model to be our primary measure of serial dependence. We show the results of the derivative of Gaussian model for completeness in Supplementary Analysis 3 (Online Supplementary Material), and find these replicate the results of the derivative of von Mises model.

Finally, again replicating the analyses of Samaha et al. (2019), we use a model-free method to quantify the size of the serial dependence effect. For each participant we took the median (signed) error on all trials with a positive orientation difference (where the orientation of the inducer stimulus or response was within the range 5° to 35°) and subtracted from this the median error on all trials with a negative orientation difference (within the range −5° to −35°). As this number represents the sum of two dependencies (those to negative orientation differences and those to positive orientation differences), we divided this value by two for the final model-free measure of serial dependence. A positive value suggests an overall attractive effect of the inducer trial, while a negative value suggests an overall repulsive effect.

### Statistical analyses

For the model-based analyses (derivatives of von Mises and Gaussian), we obtained bootstrapped CIs of the maximum amplitudes yielded by the models fit to the data split by the conditions of interest (e.g., SNR or positive evidence on the target or inducer trial to assess their statistical significance). We did this by bootstrap resampling with replacement across participants to create 10,000 surrogate datasets of the same size as the original, then fitting the model to these surrogate datasets to establish the 95% CIs. To generate a 95% CI for the difference in the size of the serial dependence effect between conditions, we used the paired differences of these bootstrapped amplitudes between conditions. For the model-free analyses, we compare the size of the bias to zero with one-tailed one-sample *t*-tests, and between conditions with paired-samples* t*-tests or repeated-measures ANOVAs. Bayes factors for these were calculated in RStudio 2024.04.2 using the *BayesFactor* package.

## Results

### Positive evidence bias

**Fig. 2:**
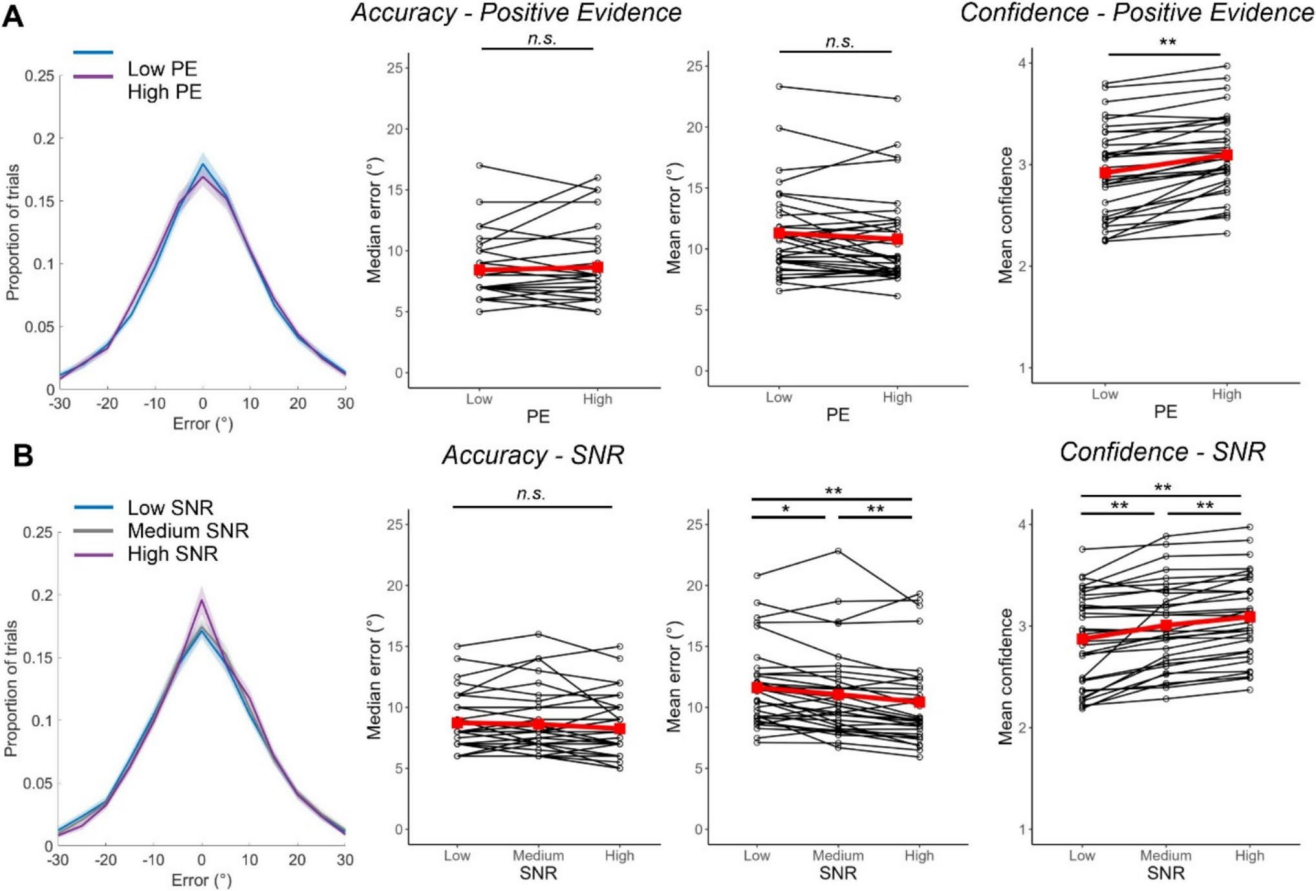
Errors and confidence across different levels of positive evidence (PE) signal-to-noise ratio (SNR). Measures of accuracy and confidence across PE (**A**) and SNR conditions (**B**). The first figure (left) shows the distribution of response errors (in bins of 5°, averaged across subjects) for each condition. Shaded areas reflect ± 1 standard error of the mean (SEM). The next figures show median error, mean error, and mean confidence rates across conditions. Black dots and lines represent individual participants’ data and red squares and lines represent group averages

We analysed absolute error on trials that were matched in SNR but had overall high positive evidence (i.e., high contrast) or low positive evidence (Fig. 2). We found no significant differences in mean error (*Low: M* = 11.30, *SD* = 3.65; *High: M* = 10.81, *SD* = 3.66; *t*[32] = 1.78, *p* = 0.085, *BF*_10_ = 0.76) or median error (*Low: M* = 8.42, *SD* = 2.55; *High: M* = 8.65, *SD* = 2.90; *t*[32] = 0.91, *p* = 0.372, *BF*_10_ = 0.27) between positive evidence conditions. There was also no difference in the precision between positive evidence conditions (*Low: M* = 24.16, *SD* = 9.87; *High: M* = 25.77, *SD* = 11.69; *t*[32] = 1.54, *p* = 0.132, *BF*_10_ = 0.55), though the log-transformed guess rate did differ (*t*[32] = 2.94, *p* = 0.006, *BF*_10_ = 6.74), being higher in the low positive evidence condition (*M* = −2.76, *SD* = 2.17) than in the high positive evidence condition (*M* = −4.27, *SD* = 2.63). It is worth noting that the log-likelihoods indicated that the model fit the high positive-evidence data (*M* = 7.99, *SD* = 39.58) significantly better than the low positive-evidence data (*M* = −1.62, *SD* = 37.33; *t*[32] = 3.06, *p* = 0.004, *BF*_10_ = 8.66), limiting the conclusions that can be drawn from the mixture model.

Importantly, and in line with the positive evidence bias, confidence was higher to high positive evidence (*M* = 3.10, *SD* = 0.41) than low positive evidence (*M* = 2.92, *SD* = 0.45) stimuli, *t*(32) = 6.87, *p* < 0.001, *BF*_10_ > 150. We looked at the proportion of the different confidence responses made between each positive evidence condition with a 2 (PE) × 4 (Confidence) repeated-measures ANOVA and found a significant interaction, *F*(2.01, 64.35) = 13.00, *p* < 0.001, *BF*_incl_ > 150 (Greenhouse–Geisser correction applied due to violation of sphericity). More low confidence responses were made to low positive evidence stimuli (‘1’ responses *p* = 0.004; ‘2’ responses *p* = 0.006) and more high confidence responses were made to high positive evidence stimuli (‘4’ responses *p* < 0.001, all *p*-values Bonferroni-corrected). Finally, following Samaha et al. (2019), we sought to demonstrate the independence of confidence and accuracy across the two levels of positive evidence by showing no relationship between the change in confidence across conditions (Low PE-High PE) and the change in accuracy across conditions for all four accuracy measures using Spearman’s rho. We found no relationship between positive evidence-related changes in confidence and positive-evidence related changes in median error (*ρ* = −0.12, *p* = 0.523, *BF*_10_ = 0.28) or log-transformed guess rate (*ρ* = −0.20, *p* = 0.272, *BF*_10_ = 0.31), anecdotal evidence for a relationship with changes in precision (*ρ* = 0.34, *p* = 0.050, *BF*_10_ = 1.06), but a significant relationship with mean error (*ρ* = −0.37, *p* = 0.037, *BF*_10_ = 7.16). Overall, we find strong evidence of the positive evidence bias in all but two measures (differences in guess rate and the relationship between changes in confidence and changes in mean error).

That accuracy did not differ between positive evidence conditions is not due to a lack of variance in accuracy: the three SNR conditions differed in mean error (*F*[1.62,51.85] = 13.78, *p* < 0.001, *BF*_10_ > 150 [Greenhouse–Geisser corrected]), with higher errors on low SNR trials (*M* = 11.63, *SD* = 3.30) compared to medium (*M* = 11.05, *SD* = 3.57; *p* = 0.025) and high (*M* = 10.43, *SD* = 3.53; *p* < 0.001) SNR trials, and higher errors on medium compared to high SNR trials (*p* = 0.009). There was anecdotal evidence that median accuracy did not differ between SNR conditions (*Low: M* = 8.73, *SD* = 2.28; *Medium: M* = 8.62, *SD* = 2.64; *High: M* = 8.26, *SD* = 2.59; *F*[1.66,52.99] = 2.15, *p* = 0.135, *BF*_10_ = 0.49 [Greenhouse–Geisser corrected]). There was good evidence that precision did differ between SNR conditions (*F*[2,64] = 7.64, *p* < 0.001, *BF*_10_ = 28.58), being greater for high SNR trials (*M* = 27.17, *SD* = 11.79) compared to low (*M* = 22.85, *SD* = 9.92; *p* = 0.007) or medium (*M* = 24.71, *SD* = 10.24; *p* = 0.036) SNR trials. Log-transformed guess rate also differed (*F*[2,64] = 7.06, *p* = 0.002, *BF*_10_ = 22.56), as the frequency of guesses was higher on medium (*M* = −2.61, *SD* = 1.90) than high SNR trials (*M* = −4.30, *SD* = 2.68; *p* = 0.003), though there was no difference between the guess rate on low SNR trials (*M* = −3.09, *SD* = 2.31) and medium (*p* = 0.740) or high (*p* = 0.077) SNR trials. This time, the log-likelihoods suggested that the mixture model fit the data equally well in all conditions (*Low: M* = −4.67, *SD* = 35.84; *Medium: M* = 5.06, *SD* = 74.64; *High: M* = 10.17, *SD* = 39.28; *F*[1.26,40.32] = 2.96, *p* = 0.084, *BF*_10_ = 0.93 [Greenhouse–Geisser corrected]). As would be expected, mean confidence also differed between the three conditions (*F*[1.13,36.22] = 29.91, *p* < 0.001, *BF*_10_ > 150 [Greenhouse–Geisser corrected]), with confidence being lowest on low SNR trials (*M* = 2.87, *SD* = 0.46), then medium (*M* = 3.01, *SD* = 0.42), then high SNR trials (*M* = 3.09, *SD* = 0.41; all *p* < 0.001; all *p*-values Bonferroni-corrected).

As some trials with larger errors were excluded from the serial dependence analysis, we checked that the trials driving the evidence for the positive evidence bias were not those being excluded. Supplementary Analysis 1 (Online Supplementary Material) reports the outcome of the above analyses using only the data used to study the serial dependence effect. Evidence for the positive evidence bias remained in the subset of data used to study the serial dependence effect.

### Effects of positive evidence on serial dependence

**Fig. 3:**
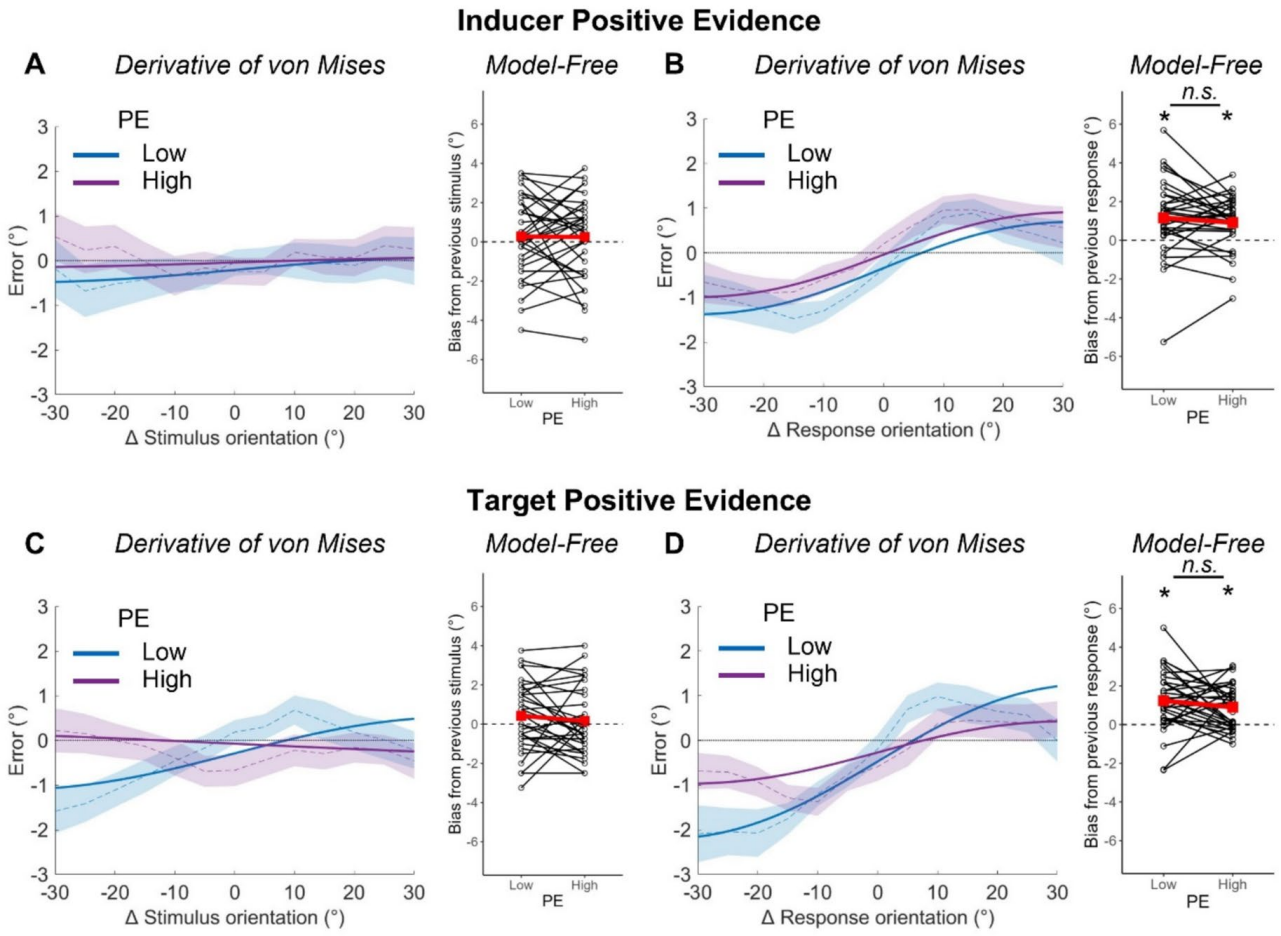
Effects of positive evidence (PE) on serial dependence to stimuli and responses. Serial dependence effect under differing levels of PE in the inducer (top, **A**, **B**) and target (bottom, **C**, **D**) using both the stimulus-based (left, **A** and **C**) and response-based (right, **B** and **D**) methods of analysis. For the derivative of von Mises analysis, positive values on the y-axis indicate clockwise errors (response-stimulus), and positive values on the x-axis indicate that the stimulus on trial *t*−1 was more clockwise than the stimulus on trial *t* (previous-current). Thick lines represent the von Mises model fit to the entire sample’s data and bounded lines represent the mean across individuals and ± 1 SEM. For the model-free analysis, positive values on the y-axis indicate an overall attractive bias to previous stimuli/responses and negative values represent an overall repulsive bias. Black dots and lines represent individual participants’ data and red squares and lines represent group averages

We first examined how serial dependence to stimuli and responses was affected by positive evidence in the inducer stimulus (Fig. 3, A and B). Using the derivative of von Mises model described above, we found no significant biases towards the inducer stimulus at either level of positive evidence in the inducer (*Low* = 0.28° [−0.85, 1.33]; *High* = 0.11° [−0.89, 0.94]), but significant biases towards the inducer response at both levels of positive evidence in the inducer (*Low* = 1.04 [0.47, 1.71] at 31.91°, 84.37% of variance explained; *High* = 0.95° [0.44, 1.63] at 31.63°, 86.66% of variance explained). These latter effects did not differ from each other (*Δ* = 0.09° [−0.50, 0.68]). The model-free method yielded no significant biases to the inducer stimulus (*Low* = 0.27° [−0.46, 1.00], *t*[32] = 0.76, *p* = 0.226, *BF*_10_ = 0.37; *High* = 0.24° [−0.51, 1.00], *t*[32] = 0.65, *p* = 0.259, *BF*_10_ = 0.33) and significant positive biases to the inducer response both when it was low positive evidence (1.16° [0.47, 1.84], *t*[32] = 3.44, *p* < 0.001, *BF*_10_ = 41.49) and high positive evidence (0.92° [0.44, 1.40], *t*[32] = 3.91, *p* < 0.001, *BF*_10_ = 130.60), and these did not differ from one another (*t*[32] = 0.95, *p* = 0.349, *BF*_10_ = 0.28).

We next considered how serial dependence was affected by positive evidence in the target stimulus (Fig. 3, C and D). Using the derivative of von Mises model we observed a significant positive bias towards the inducer stimulus for low positive-evidence target stimuli (0.82° [0.08, 1.60] at 39.04°, 72.28% of variance explained) but not high positive-evidence target stimuli (−0.21° [−0.99, 0.57]), and found that these effects were significantly different to one another (*Δ* = 1.03° [0.26, 1.83]). We found a significant positive bias towards the inducer response at both levels of positive evidence in the target (*Low* = 1.73° [1.06, 2.49] at 36.04°, 86.61% of variance explained; *High* = 0.70° [0.21, 1.28] at 32.38°, 81.78% of variance explained), which was again larger for low positive-evidence targets (*Δ* = 1.03° [0.35, 1.73]). Using the model-free method yielded no significant positive biases to the inducer stimulus at any level of positive evidence in the target (*Low* = 0.42° [−0.20, 1.05], *t*(32) = 1.39, *p* = 0.087, *BF*_10_ = 0.81; *High* = 0.16° [−0.46, 0.78], *t*[32] = 0.52, *p* = 0.302, *BF*_10_ = 0.29) and a significant positive skew towards the orientation of the inducer response for low positive-evidence targets (1.41° [0.75, 2.06], *t*[32] = 4.37, *p* < 0.001, *BF*_10_ > 150) and high positive-evidence targets (0.90° [0.49, 1.31], *t*[32] = 4.43, *p* < 0.001, *BF*_10_ > 150). These two effects again did not differ from each other (*t*[32] = 1.50, *p* = 0.144, *BF*_10_ = 0.51).

To summarise, we find no evidence of a modulatory effect of positive evidence in the inducer stimulus but some evidence that serial dependence was modulated by positive evidence in the target stimulus, being larger on trials with low positive-evidence target stimuli.

### Effects of signal-to-noise ratio on serial dependence

**Fig. 4:**
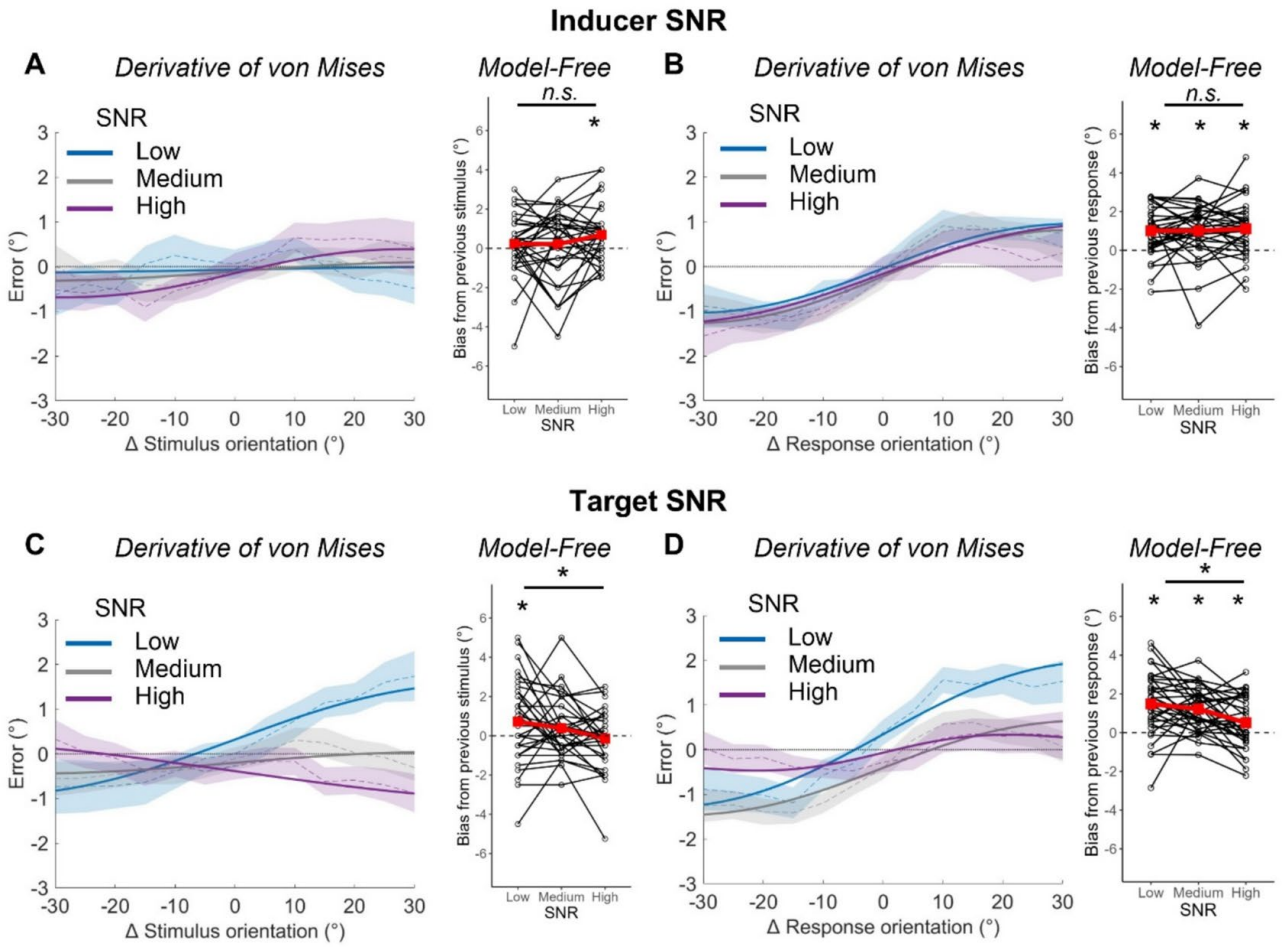
Effects of signal-to-noise ratio (SNR) on serial dependence to stimuli and responses. Serial dependence effect under differing levels of SNR in the inducer (top, A, B) and target (bottom, C, D) using both the stimulus-based (left, A and C) and response-based (right, B and D) methods of analysis. For the derivative of von Mises analysis, positive values on the y-axis indicate clockwise errors (response-stimulus), and positive values on the x-axis indicate that the stimulus on trial *t*−1 was more clockwise than the stimulus on trial *t* (previous-current). Thick lines represent the von Mises model fit to the entire sample’s data and bounded lines represent the mean across individuals and ± 1 SEM. For the model-free analysis, positive values on the y-axis indicate an overall attractive bias to previous stimuli/responses and negative values represent an overall repulsive bias. Black dots and lines represent individual participants’ data and red squares and lines represent group averages

We considered how serial dependence was modulated by SNR in the inducer stimulus (Fig. 4, A and B). The derivative of von Mises model yielded no significant biases to the previous stimulus at any level of SNR in the inducer (*Low* = 0.07° [−0.70, 0.72]; *Medium* = 0.21° [−0.71, 0.99]; *High* = 0.54° [−0.11, 1.26]) but significant positive biases to the previous response at all levels of SNR in the inducer (*Low* = 1.00 [0.60, 1.51] at 32.55°, 88.31% of variance explained; *Medium* = 1.05 [0.55, 1.62] at 32.33°, 86.79% of variance explained; *High* = 1.11° [0.45, 1.89] at 37.59°, 89.02% of variance explained). These amplitudes did not differ from one another (*ΔLow-Medium* = −0.05° [−0.54, 0.53]; *ΔLow-High* = −0.11° [−0.83, 0.62]; *ΔMedium-High* = −0.06° [−0.70, 0.54]). In the model-free analysis there was a significant positive bias to the inducer stimulus when it had a high SNR (0.69° [0.17, 1.21], *t*[32] = 2.69, *p* = 0.006, *BF*_10_ = 7.79), but an ANOVA suggested this did not differ from the non-significant biases following medium SNR (0.21° [−0.45, 0.87], *t*[32] = 0.66, *p* = 0.258, *BF*_10_ = 0.33) and low SNR (0.23° [−0.32, 0.77], *t*[32] = 0.85, *p* = 0.200, *BF*_10_ = 0.41) inducer stimuli (*Δ: F*[2,64] = 1.45, *p* = 0.243, BF_*incl*_ = 0.30). There was also a significant positive bias to the inducer response for high (1.11° [0.61, 1.62], *t*[32] = 4.50, *p* < 0.001, *BF*_10_ > 150), medium (1.00° [0.50, 1.51], *t*[32] = 4.02, *p* < 0.001, *BF*_10_ > 150), and low SNR inducer stimuli (1.01° [0.57, 1.45], *t*[32] = 4.63, *p* < 0.001, *BF*_10_ > 150) but an ANOVA suggested these did not differ from one another (*F*[2,64] = 0.13, *p* = 0.878, *BF*_*incl*_ = 0.10).

When considering the role of SNR in the target stimulus (Fig. 4, C and D), the derivative of von Mises model yielded a significant positive bias towards the inducer stimulus only for low SNR target stimuli (1.25° [0.34, 2.24] at 41.57°, 98.29% of variance explained). This was larger than the non-significant effects for medium SNR (0.24° [−0.45, 0.90]; *Δ* = 1.01° [0.23, 1.89]) and high SNR (−0.60° [−1.35, 0.89]; *Δ* = 1.85° [1.16, 2.55]) target stimuli. We found significant positive biases towards the inducer response when the SNR of the target stimulus was low (1.63° [0.93, 2.41] at 36.98°, 93.70% of variance explained) or medium (1.06° [0.56, 1.63] at 34.17°, 86.82% of variance explained). The magnitude of the effect did not differ between these two conditions (*Δ* = 0.57° [−0.15, 1.35]), but they were both larger than the non-significant function for high SNR target stimuli (0.40° [−0.23, 1.94]; *ΔMedium-High* = 0.66° [0.12, 1.17]; *ΔLow-High* = 1.23° [0.57, 1.88]). In the model-free analysis there was a significant positive bias towards the inducer stimulus when the target SNR was low (0.72° [−0.04, 1.48], *t*[32] = 1.94, *p* = 0.031, *BF*_10_ = 1.66) though the evidence for this was anecdotal, and there was only anecdotal evidence for the overall effect of SNR that would indicate this differed from the non-significant biases when the target stimulus was medium (0.39° [−0.16, 0.93], *t*[32] = 1.45, *p* = 0.079, *BF*_10_ = 0.88) or high SNR (−0.14° [−0.70, 0.41], *t*[32] = 0.53, *p* = 0.700, *BF*_10_ = 0.13; *Δ: F*[2,64] = 3.70, *p* = 0.030, BF_*incl*_ = 1.70). There was a significant positive skew towards the orientation of the inducer response at low (1.48° [0.90, 2.07], *t*[32] = 5.19, *p* < 0.001, *BF*_10_ > 150), medium (1.22° [0.83, 1.62], *t*[32] = 6.30, *p* < 0.001, *BF*_10_ > 150), and high (0.51° [0.04, 0.99], *t*[32] = 2.19, *p* = 0.018, *BF*_10_ = 2.94) levels of SNR in the target. These differed significantly (*F*[2,64] = 7.45, *p* = 0.001, *BF*_*incl*_ = 27.80), with the effect for high SNR target stimuli being significantly smaller than that for medium (*p* = 0.009) and low (*p* = 0.006) SNR targets.

We find no effect of the SNR of the inducer stimulus on serial dependence. However, we consistently observed greater biases to previous stimuli and responses when the SNR of the target stimulus was low compared to high.

### Effects of subjective confidence on serial dependence

**Fig. 5:**
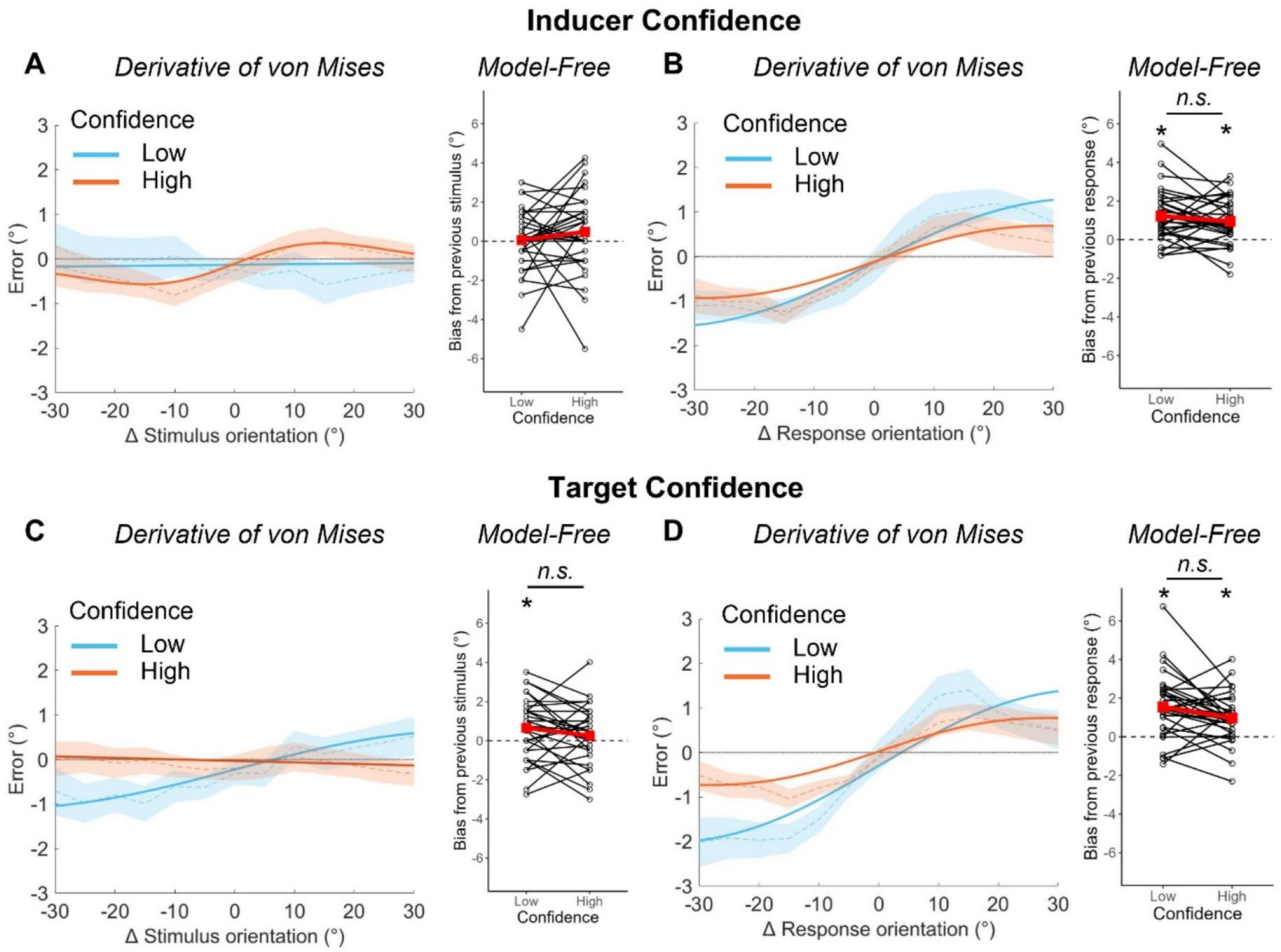
Effects of subjective confidence on serial dependence to stimuli and responses. Serial dependence effect under differing levels of confidence in the inducer (top, **A**, **B**) and target (bottom, **C**, **D**) using both the stimulus-based (left, **A** and **C**) and response-based (right, **B** and **D**) methods of analysis. For the derivative of von Mises analysis, positive values on the y-axis indicate clockwise errors (response-stimulus), and positive values on the x-axis indicate that the stimulus on trial *t*−1 was more clockwise than the stimulus on trial *t* (previous-current). Thick lines represent the von Mises model fit to the entire *s*ample’s data and bounded lines represent the mean across individuals and ± 1 SEM. For the model-free analysis, positive values on the y-axis indicate an overall attractive bias to previous stimuli/responses and negative values represent an overall repulsive bias. Black dots and lines represent individual participants’ data and red squares and lines represent group averages

The purpose of the positive evidence manipulation was to allow us to examine the effect of different levels of response confidence independently from accuracy. However, as we found little evidence for an effect of positive evidence in the inducer stimulus, we next asked if serial dependence was modulated by raw subjective confidence in the previous response (Fig. 5, A and B). We split the data into ‘Low’ and ‘High’ confidence responses based on whether the confidence response was smaller or larger than the participant’s mean confidence response, as done by Samaha et al. (2019). We report the findings split by each individual confidence response in Supplementary Analysis 4 (Online Supplementary Material).

We found no serial dependence to the previous stimulus at either level of confidence in the inducer response (*Low* = 0.05° [0.10, 1.66]; *High* = 0.46° [−0.48, 1.13]) but significant serial dependence to the inducer response in both cases (*Low* = 1.45° [0.97, 1.96] at 35.92°, 93.85% of variance explained; *High* = 0.81° [0.33, 1.52] at 29.10°, 79.65% of variance explained [derivative of von Mises]). These significant effects did not differ from one another (*Δ* = 0.64° [−0.07, 1.21]). The model-free method showed no significant biases to the inducer stimulus (*Low* = 0.05° [−0.49, 0.60], *t*[32] = 0.20, *p* = 0.422, *BF*_10_ = 0.19; *High* = 0.48° [−0.24, 1.20], *t*[32] = 1.35, *p* = 0.093, *BF*_10_ = 0.43) and a significant bias to the inducer response regardless of subjective confidence on the inducer trial (*Low* = 1.22° [0.76, 1.68], *t*[32] = 5.38, *p* < 0.001, *BF*_10_ > 150; *High* = 0.94° [0.53, 1.36], *t*[32] = 4.64, *p* < 0.001, *BF*_10_ > 150; *Δ: t*[32] = 1.19, *p* = 0.242, *BF*_10_ = 0.36).

Finally, we considered if serial dependence might differ based on the confidence on the target trial (Fig. 5, B and C). Serial dependence may be largest for low-confidence target trials, as these trials are more likely to be low SNR and low positive evidence. Alternatively, if serial dependence functions to decrease the noise experienced in the percept, confidence responses maybe be inflated to targets that have been more influenced by serial biases. The derivative of von Mises model showed greater serial dependence to the previous stimulus when a low confidence response was made on the target trial (0.89° [0.10, 1.66] at 41.27°, 94.74% of variance explained) compared to the non-significant effect when a high confidence response was made (−0.16° [−0.92, 0.55]; *Δ* = 1.05° [0.18, 1.83]). The bias to the previous response was significant at both levels of confidence on the target trial (*Low* = 1.73° [1.11, 2.41] at 36.45°, 92.21% of variance explained; *High* = 0.75° [0.36, 1.33] at 31.81°, 82.15% of variance explained) and was still larger on low confidence target trials (*Δ* = 0.98° [0.25, 1.62]). The model-free method showed a significant bias to the previous stimulus on low confidence trials only (0.65° [0.10, 1.21], *t*[32] = 2.40, *p* = 0.011, *BF*_10_ = 2.21), but this did not differ from the non-significant effect on high confidence trials (0.25° [−0.29, 0.79], *t*[32] = 0.94, *p* = 0.176, *BF*_10_ = 0.28; *Δ: t*[32] = 1.49, *p* = 0.147, *BF*_10_ = 0.51). There was a significant bias to the previous response on low (1.54° [0.95, 2.13], *t*[32] = 5.34, *p* < 0.001, *BF*_10_ > 150) high (0.97° [0.53, 1.41], *t*[32] = 4.47, *p* < 0.001, *BF*_10_ > 150) confidence trials. This was significantly greater when there was low subjective confidence on the target trial (*t*[32] = 2.06, *p* = 0.048, *BF*_10_ = 1.19).

To summarise, we found no effect of subjective confidence in the inducer response on serial dependence to either stimuli or response. However, serial dependence effects tended to be larger on trials where responses were made with low confidence.

## Discussion

We examined how two sources of uncertainty – in stimuli and the decisions made about them – interacted at different times to affect serial dependence. We found that serial dependence was greatest for more uncertain target stimuli, including those that were low SNR, low positive evidence, or were responded to with low subjective confidence. This suggests that more uncertain stimuli induced a greater bias towards previously seen orientations. This aligns with previous studies showing that serial dependence is greater when faced with uncertain visual input (Cicchini et al., 2018; Clifford et al., 2018; Gallagher & Benton, 2022; van Bergen & Jehee, 2019) and the idea that serial dependence functions to counteract uncertainty.

Samaha et al. (2019) reported greater serial dependence following high positive-evidence inducers, and suggested this means that decisions that are experienced with low confidence are less likely to influence future decisions. Suárez-Pinilla et al. (2018) also report an effect of decision confidence on the inducer trial on serial dependence in the perception of numerosity, such that there was a greater bias towards high confidence decisions, and no effect of confidence on the target trial. We found that serial dependence was not modulated by the positive evidence or the raw subjective confidence on the inducer trial, suggesting that more certain previous decisions were not more likely to influence subsequent decisions, and that the previously reported effects of positive evidence may not generalise across paradigms.

Serial dependence was sensitive instead to uncertainty in the target trial. Greater serial dependence was observed on trials that were responded to with low subjective confidence, which are likely to be those that were low positive evidence or low SNR (as suggested by the analyses in the section *Positive evidence bias*). Greater serial dependence was also observed when the target had low positive evidence, which was operationalised as low contrast. This aligns with the finding that serial dependence in judgements of positions is greater to low-contrast stimuli (Manassi et al., 2018), though in this study the comparison was between tasks in which all stimuli were either high contrast or low contrast, so whether their effect was being driven by the contrast of the inducer or stimulus (or both) is unclear. Greater serial dependence was observed when the target stimulus had a low SNR, adding to the wealth of findings that serial dependence is greater under perceptual uncertainty (Ceylan et al., 2021; Cicchini et al., 2017, 2018; Clifford et al., 2018; Fulvio et al., 2023; Gallagher & Benton, 2022; Manassi & Whitney, 2022), and that this modulation is specific to the target stimulus (Cicchini et al., 2018; Fulvio et al., 2023; Gallagher & Benton, 2022). The effect of SNR in the target stimulus was also the most robust across different methods of analyses (stimulus- and response-based, model-based and model-free), emphasising the importance of perceptual information in serial dependence.

The predictions of this experiment were that post-perceptual history might exert an effect on perception – i.e., that the source of serial dependence is decisional and the site perceptual. That greater serial dependence did not occur to more certain decisions does not align with this prediction, nor with other models describing serial dependence as a skew towards previous decisions. For example, Pascucci and colleagues (2019) propose that responses are subject to both an assimilative skew towards the previous decision made, and a repulsive neuronal response (adaptation) away from the previous sensory input. The resulting decision is the sum of these two distributions; assimilative serial dependence is observed where adaptation is low (e.g., with low-contrast and briefly presented stimuli) and the distribution of the decision inertia is narrow (i.e., where decisions are made with more certainty). Here we observe serial dependence even when stimuli were presented briefly (33 ms) and we find no effect of uncertainty in previous decisions. Our findings are also at odds with popular Bayesian accounts of serial dependence (e.g., Cicchini et al., 2018; Fritsche et al., 2020; van Bergan & Jehee, 2019), as while more uncertain visual inputs induced a greater reliance on prior information, we did not find the other effect predicted by the Bayesian account: that perception should be less influenced by prior inputs that were more uncertain, either at the level of the stimulus itself or the decision made about it.

That serial dependence was greater to previous responses over previous stimuli could be construed as evidence for a decisional account. However, the response is likely to be a better representation of the percept experienced by the stimulus than the stimulus itself is (Zhang & Alais, 2020). This interpretation is difficult to find evidence for when the two are so closely correlated – the inducer response will be similar to the inducer stimulus where task performance is good. However, presenting a target stimulus that is the midpoint between the inducer stimulus and inducer response reveals that the bias occurs towards the response and away from the stimulus (Gallagher & Benton, 2024).

It is worth noting that some measures of the positive evidence bias were not significant in our manipulation check, as the guess rate differed between conditions where it ideally should not, and we did find a relationship between positive evidence-related changes in confidence and positive evidence-related changes in mean error. Additionally, the observed Bayes Factors suggested an absence of evidence for the difference in mean error and precision between conditions, rather than evidence for no difference. However, these findings were related to accuracy – we consistently found strong evidence that confidence differed between the two levels of positive evidence, yet this had no effect on serial dependence to high- and low-positive evidence inducers. Another important difference in our study was that we used the average values of stimulus contrast for each positive evidence condition reported by Samaha et al. (2019) rather than taking these from each individual participant’s threshold as the authors of the previous study did. Our participants may have found the task too easy overall, perhaps evidenced by the slightly higher overall confidence that we observed, and the smaller errors in adjustment responses. However, the smaller errors made our results more robust against the exclusion of high-error trials (see Supplementary Analysis 5, Online Supplementary Material). We also know that the positive evidence conditions differed from one another enough to induce some difference, as we found consistent effects of positive evidence in the target stimulus. We interpret this as further evidence of the serial dependence being larger to more uncertain target stimuli – low positive-evidence targets, which were lower in contrast and associated with a higher frequency of guess responses, induced a greater reliance on previous information.

## Conclusion

We found that serial dependence was reliably facilitated by uncertainty in the target stimulus, but not by decisional or stimulus uncertainty in the inducer. That the effect was not modulated by uncertainty in prior visual input or decisions does not align with Bayesian accounts of serial dependence, and that there was no clear influence of decisional uncertainty suggests that serial dependence may not reflect a skew towards previous decisions.

## Supplementary Information

Below is the link to the electronic supplementary material.

Supplementary file1 (DOCX 291 KB)

## Data Availability

Raw datasets are available in the Open Science Framework (OSF) repository, https://osf.io/kvy4c/. None of the experiments was preregistered.

## Code availability

Experimental code is available in the Open Science Framework (OSF) repository, https://osf.io/kvy4c/
