# Supplementary material for: Effects of perceptual and decisional uncertainty on serial dependence in orientation perception
Source: Atten Percept Psychophys. 2025 Mar 3;87(3):909–21. doi: 10.3758/s13414-025-03034-5 (PMC11965219; doi:10.3758/s13414-025-03034-5)
Supplement: Supplementary file 1 — Supplementary file1 (DOCX 291 KB) [file 13414_2025_3034_MOESM1_ESM.docx]

**Supplementary Analyses**

**Supplementary Analysis 1**

**Figure S1**

*Errors and Confidence Across Different Levels of Positive Evidence Signal-to-Noise Ratio*

*After Trial Exclusion*


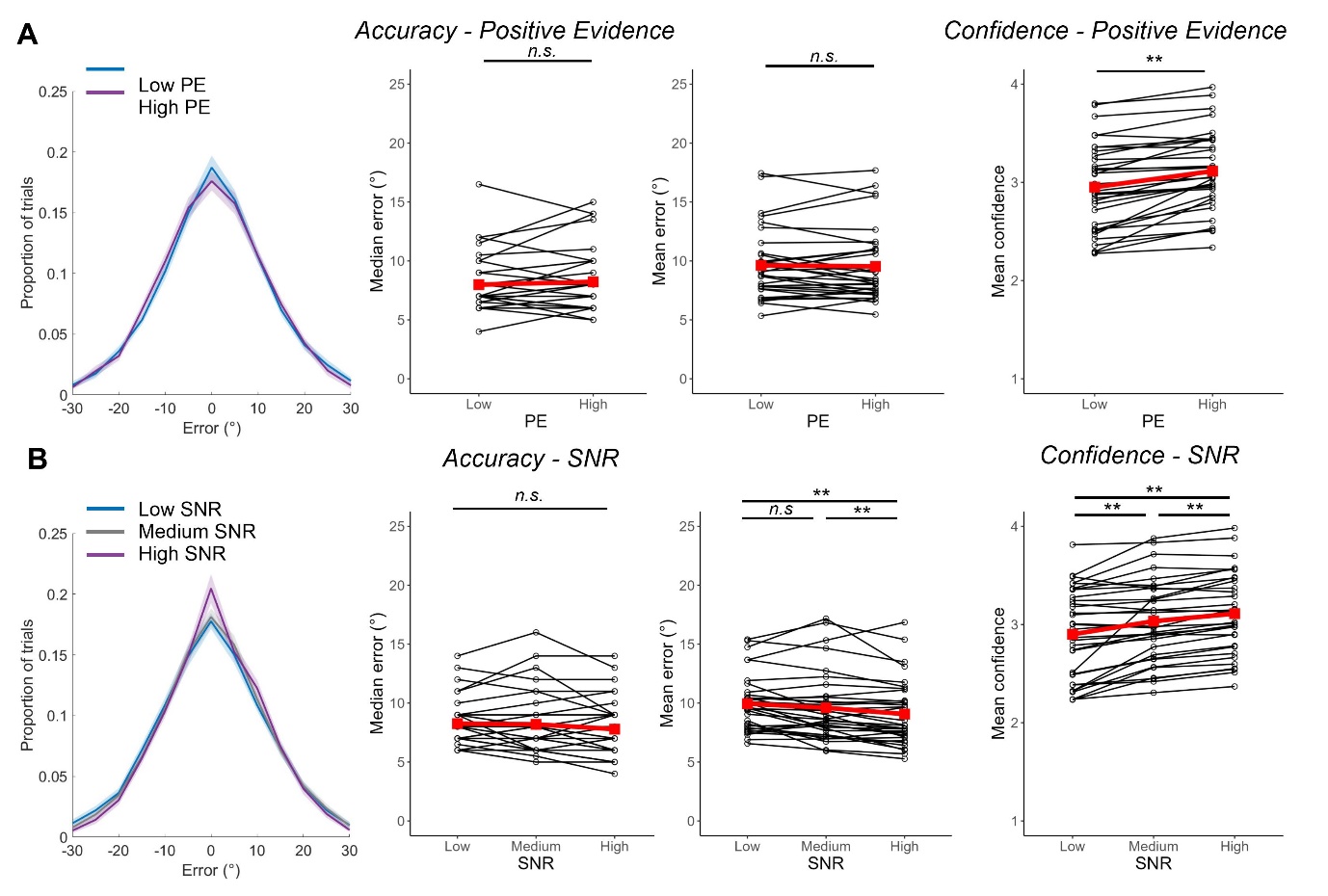


*Note.* Measures of accuracy and confidence across positive evidence (A) and SNR conditions (B). The first figure (left) shows the distribution of response errors (in bins of 5°, averaged across subjects) for each condition. Shaded areas reflect ±1 standard error of the mean (SEM). The next figures show median error, mean error, and mean confidence rates across conditions. Black dots and lines represent individual participants’ data and red squares and lines represent group averages. (PE = Positive Evidence).

Once trials with outlying response times and accuracy had been removed, we still found moderate evidence for no differences in mean (*Low: M* = 9.63, *SD* = 2.91; *High: M* = 9.54, *SD* = 3.08; *t*[32] = 0.42, *p* = .678, *BF*_10_ = 0.20) or median (*Low: M* = 7.99, *SD* = 2.45; *High: M* = 8.23, *SD* = 2.71; *t*[32] = 0.91, *p* = .372, *BF*_10_ = 0.27) error between positive evidence conditions, and anecdotal evidence for no difference in precision (*Low: M* = 28.90, *SD* = 14.75; *High: M* = 31.28, *SD* = 15.36; *t*[32] = 1.61, *p* = .116, *BF*_10_ = 0.60). We again observed anecdotal evidence for a difference in log-transformed guess rate (*t*[32] = 2.37, *p* = .024, *BF*_10_ = 2.08), such that guess frequency was higher in the low positive evidence condition (*M* = -7.12, *SD* = 0.69) than in the high positive evidence condition (*M* = -7.35, *SD* = 0.42). This time, the mixture model fit the data equally well for both conditions (*Low: M* = 21.53, *SD* = 34.88; *High: M* = 26.07, *SD* = 37.29; *t*[32] = 1.67, *p* = .105, *BF*_10_ = 0.65)

Confidence responses were higher to high positive evidence (*M* = 3.12, *SD* = 0.41) than low positive evidence (*M* = 2.96, *SD* = 0.45) stimuli, *t*(32) = 6.45, *p* < .001, *BF*_10_ > 150. The interaction of positive evidence and confidence (*F*(1.96, 62.68) = 11.92, *p* < .001, *BF*_10_ > 150 [Greenhouse-Geisser corrected]) showed the same significant differences: (1 responses *p* = .019; 2 responses *p* = .006; 3 responses *p* = 1.000; 4 responses *p* < .001, all *p*-values Bonferroni-corrected). There was anecdotal evidence for no relationship between positive evidence-related changes in confidence and positive-evidence related changes in median error (*ρ* = -0.31, *p* = .084, *BF*_10_ = 0.79), inconclusive evidence about the relationship with log-transformed guess rate (*ρ* = -0.32, *p* = .067, *BF*_10_ = 1.17), and significant relationships with precision (*ρ* = 0.44, *p* = .011, *BF*_10_ = 6.17), and mean error (*ρ* = -0.36, *p* = .040, *BF*_10_ = 4.06). Overall, while there was more of a relationship between confidence and accuracy in the two positive evidence conditions in the subset of trials used for the serial dependence analysis, most checks still yielded signs of the positive evidence bias.

Looking at the effect of SNR on accuracy, we found that the three SNR conditions differed in mean error (*F*[1.68,46.24] = 0.08, *p* < .001, *BF*_10_ > 150 [Greenhouse-Geisser corrected]), with errors being larger on low SNR trials (*M* = 9.95, *SD* = 2.41) and medium SNR trials (*M* = 9.60, *SD* = 2.93) compared to high SNR trials (*M* = 9.07, *SD* = 2.79; Low-High *p* = .004, Low-Med *p* = .011). The effect of SNR on median accuracy was non-significant and only anecdotal in the Bayesian analyses (*Low:* *M* = 8.26, *SD* = 2.08; *Medium:* *M* = 8.20, *SD* = 2.63; *High:* *M* = 7.79, *SD* = 2.46; *F*[2,64] = 2.37, *p* = .102, *BF*_10_ = 1.12). Precision differed between SNR conditions (*F*[2,64] = 13.09, *p* < .001, *BF*_10_ > 150), being smaller for low SNR trials (*M* = 22.85, *SD* = 9.92) compared to medium (*M* = 29.63, *SD* = 14.12; *p* < .001) or high (*M* = 27.17, *SD* = 11.79; *p* = .007) SNR trials. For guess rate (*F*[2,64] = 48.33, *p* < .001, *BF*_10_ > 150), the medium SNR condition (*M* = -7.37, *SD* = 1.09) demonstrated the lowest log-transformed frequency of guess responses compared to the low (*M* = -3.09, *SD* = 2.31; *p* < .001) and high (*M* = 4.30, *SD* = 2.68; *p* < .001) SNR conditions. However, the model also fit the data best in the medium SNR data (*M* = 46.65, *SD* = 70.28) compared to high (*M* = 10.17, *SD* = 39.28) or low (*M* = -4.67, *SD* = 35.84) SNR (*F*[1.32,42.27] = 44.67, *p* < .001, *BF*_10_ > 150; all comparisons *p* < .001). Mean confidence also differed between the three conditions (*F*[1.14,36.42] = 29.48, *p* < .001, *BF*_10_ > 150 [Greenhouse-Geisser corrected]), with confidence being lowest on low SNR trials (*M* = 2.90, *SD* = 0.46), then medium (*M* = 3.03, *SD* = 0.42), then high SNR trials (*M* = 3.11, *SD* = 0.41; all *p* < .001).

**Supplementary Analysis 2**

Response-based analyses of serial dependence may yield spurious “serial dependence-like” effects that do not actually depend on trial order. This is because the inducer and target response are both subject to the same idiosyncratic response biases (e.g., around the cardinal axes; Gallagher & Benton, 2022; 2024; Manassi et al., 2018; Pascucci et al., 2019). These spurious effects can be observed by analysing data that cannot possibly include an effect of the previous response on the current response – in our case, following previous literature (Gallagher & Benton, 2022; 2024), we used data in which the order of the trials had been shuffled. We reversed the order of even-numbered trials by swapping the first even-numbered trial with the final even-numbered trial (e.g., trials number 2 and 520), the second even-numbered trial with the penultimate even-numbered trial (trials number 4 and 518), and so on for all even trials. We then analysed the shuffled data using the methods of analysis described in the main text. A significant “serial dependence-like” effect was evident in these shuffled trials in almost all levels of positive evidence and SNR in the inducer and target, or else the model yielded non-significant but still numerically large spurious effects (Tables S1-S3, left column). These effects are spurious as they cannot reflect any actual influence of the inducer trial on the target, as the trials labelled as such in this analysis were not actually shown in sequence. This suggests that a procedure to remove the effect of idiosyncratic response biases was necessary.

Previous literature suggests that these response biases take on the shape of a sum of three sinusoids when errors are plotted against the orientation of the target stimulus, and that residualizing the errors made using this function therefore removes the effect of response biases on the data (Gallagher et al., 2022; 2024; Pascucci et al., 2019). For each participant, we fit a sum of three sinusoids to the relationship between errors and orientation. We did this separately for each level of uncertainty in the target trial: low SNR (RMSE *M* = 7.07, *SD* = 2.38), medium SNR with low positive evidence (RMSE *M* = 7.05, *SD* = 3.20), medium SNR with high positive evidence (RMSE *M* = 6.27, *SD* = 2.88), and high SNR (RMSE *M* = 6.56, *SD* = 2.30). Fitting the function within each level of uncertainty separately is important as the strength and tuning of these responses biases changes with uncertainty (Gallagher et al., 2022; Tomassini et al., 2010). We subtracted the fit of the model from the response on each trial, then split the data into our conditions of interest and analysed the residualised data with our three analysis methods. Tables S1-S3 (right column) show the size of the effect in the shuffled data post-residualisation.

**Table S1**

*Spurious Effects in the Shuffled Data Before and After Residualisation with the Sum of Three Sinusoids (Derivative of von Mises Analysis).*

|  |  |  | Pre-Residualisation | Post-Residualisation |
| --- | --- | --- | --- | --- |
|  |  |  | Amplitude (°) [95% CI] | |
| Positive Evidence | Inducer | Low | 1.64 [0.94, 2.22] | 0.35 [-0.15, 0.80] |
|  |  | High | 1.82 [0.64, 2.22] | -0.05 [-0.60, 0.36] |
|  | Target | Low | 1.61 [0.50, 2.45] | -0.12 [-0.47, 0.72] |
|  |  | High | 1.56 [-0.17, 2.52] | -0.05 [-0.57, 0.48] |
| SNR | Inducer | Low | 1.89 [0.97, 2.67] | 0.03 [-0.32, 0.68] |
|  |  | Medium | 1.56 [0.92, 2.21] | 0.02 [-0.28, 0.37] |
|  |  | High | 1.32 [-0.12, 2.26] | 0.03 [-0.49, 0.49] |
|  | Target | Low | 1.28 [0.33, 2.16] | 0.20 [-0.21, 0.45] |
|  |  | Medium | 1.62 [0.88, 2.29] | -0.07 [-0.34, 0.28] |
|  |  | High | 1.71 [0.98, 2.39] | 0.22 [-0.20, 0.56] |

**Table S2**

*Spurious Effects in the Shuffled Data Before and After Residualisation with the Sum of Three Sinusoids (Derivative of Gaussian Analysis).*

|  |  |  | Pre-Residualisation | Post-Residualisation |
| --- | --- | --- | --- | --- |
|  |  |  | Amplitude (°) [95% CI] | |
| Positive Evidence | Inducer | Low | 1.33 [0.85, 1.67] | 0.41 [0.06, >1000] |
|  |  | High | 1.23 [0.69, 1.57] | -0.05 [0.00, 0.73] |
|  | Target | Low | 1.23 [0.68, 1.57] | 0.10 [0.04, >1000] |
|  |  | High | 1.24 [0.53, 1.71] | 0.03 [-0.33, 0.66] |
| SNR | Inducer | Low | 1.46 [0.92, 1.72] | 0.00 [<1000, 0.76] |
|  |  | Medium | 1.27 [0.84, 1.58] | 0.05 [<1000, 0.44] |
|  |  | High | 1.09 [0.43, 1.51] | 0.14 [-0.18, 0.90] |
|  | Target | Low | 1.13 [0.55, 1.56] | 0.03 [-0.48, 0.64] |
|  |  | Medium | 1.29 [0.82, 1.59] | 0.05 [-0.37, 0.39] |
|  |  | High | 1.34 [0.89, 1.63] | 0.01 [-0.01, 0.66] |

**Table S3**

*Spurious Effects in the Shuffled Data Before and After Residualisation with the Sum of Three Sinusoids (Model-Free Analysis).*

|  |  |  | Pre-Residualisation | Post-Residualisation |
| --- | --- | --- | --- | --- |
|  |  |  | Bias (°) [95% CI], *p*-value, BF_10_ | |
| Positive Evidence | Inducer | Low | 0.52 [0.00, 1.04], .024, 2.28 | 0.27 [-0.16, 0.70], .107, 0.69 |
|  |  | High | 0.20 [-0.33, 0.72], .226, 0.37 | 0.08 [-0.36, 0.51], .363, 0.25 |
|  | Target | Low | 0.42 [0.06, 0.79], .013, 3.97 | 0.19 [-0.15, 0.53], .128, 0.59 |
|  |  | High | 0.37 [-0.27, 1.01], .124, 0.61 | -0.04 [-0.35, 0.27], .613, 0.15 |
| SNR | Inducer | Low | 0.45 [-0.02. 0.93], .030, 1.93 | 0.25 [-0.06, 0.56], .055. 1.17 |
|  |  | Medium | 0.44 [0.04, 0.84], .016, 3.27 | 0.07 [-0.19, 0.33], .287, 0.31 |
|  |  | High | 0.47 [0.01, 0.93], .023, 2.41 | 0.10 [-0.24, 0.45], .273, 0.32 |
|  | Target | Low | 0.58 [-0.06, 1.22], .036, 1.65 | 0.23 [-0.13, 0.58], .101, 0.43 |
|  |  | Medium | 0.44 [0.09, 0.79], .008, 5.87 | 0.11 [-0.14, 0.35], .191, 0.43 |
|  |  | High | 0.32 [-0.24, 0.87], .126, 0.60 | 0.24 [-0.14, 0.62], .100, 0.73 |

For the derivative of von Mises model and the model-free method, the spurious effect was no longer significant in the residualised data (Tables S1 and S3). However, for the derivative of Gaussian model (Table S2), significant effects still remained in a small number of conditions. We therefore consider the derivative of Gaussian model to be a secondary measure and report the results from this analysis in Supplementary Analysis 3.

**Supplementary Analysis 3**

Table S4 shows the outputs of the derivative of Gaussian model for all the analyses reported in the main text. We again found no significant biases to the inducer stimulus at either level of positive evidence in the inducer but significant biases to the inducer response in both cases, and these did not differ from each other (*Δ* = 0.09° [-0.50, 0.68]). There was a significant bias towards inducer stimuli for low positive evidence targets only, though this did not differ from the non-significant effect for high positive evidence targets (*Δ* = 0.57° [-1.15, 946.34]). There were significant biases towards the previous response for both levels of positive evidence in the target, and this was larger for low positive evidence target stimuli (*Δ* = 0.75° [0.24, 1.26]).

**Table S4**

*Effects of Positive Evidence, SNR, and Subjective Confidence in the Inducer and Target Stimulus on Serial Dependence to Stimuli and Responses Using the Derivative of Gaussian Model.*

|  |  |  | Stimulus-Based | Response-Based |
| --- | --- | --- | --- | --- |
|  |  |  | Amplitude (°) [95% CI] | |
| Positive Evidence | Inducer | Low | 0.26 [-0.11, 2.96] | 1.06 [0.56, 1.58] |
|  |  | High | 0.00 [-0.48, 0.89] | 0.94 [0.50, 1.40] |
|  | Target | Low | 0.69 [0.06, 1.58] | 1.49 [0.99, 2.06] |
|  |  | High | 0.12 [-0.14, 1.70] | 0.74 [0.38, 1.16] |
| SNR | Inducer | Low | 0.09 [-0.29, >1000] | 0.96 [0.64, 1.32] |
|  |  | Medium | 0.00 [-0.27, >1000] | 1.03 [0.64, 1.45] |
|  |  | High | 0.58 [0.11, 1.26] | 0.94 [0.49, 1.61] |
|  | Target | Low | 1.03 [0.34, 2.24] | 1.39 [0.89, 2.02] |
|  |  | Medium | 0.30 [<-1000, 0.82] | 1.01 [0.64, 1.43] |
|  |  | High | 0.02 [<-1000, >1000] | 0.48 [0.06, 0.96] |
| Confidence | Inducer | Low | -0.07 [0.10, 1.66] | 1.21 [0.84, 1.82] |
|  |  | High | 0.49 [0.00, 1.01] | 0.79 [0.45, 1.29] |
|  | Target | Low | 0.74 [0.58, 1.22] | 1.44 [1.11, 2.41] |
|  |  | High | 0.15 [0.12, 0.84] | 0.75 [0.36, 1.33] |

For the effect of SNR in inducer stimuli, there was serial dependence to the previous stimulus for high SNR inducer stimuli only, but this did not differ from the non-significant amplitudes following low (*Δ* = -0.49° [-1.06, >1000]) or medium (*Δ* = -0.58° [-0.91, >1000]) SNR inducers. There were significant serial dependencies to the previous response in all conditions and these did not differ from one another (*ΔLow-Medium* = -0.07° [-0.43, 0.38]; *ΔLow-High* = 0.02° [-0.55, 0.44]; *ΔMedium-High* = 0.09° [-0.44, 0.46]). There was a significant positive bias to previous stimuli only for low SNR targets and this was larger than the non-significant effects for medium SNR (*Δ* = 0.73° [0.06, >1000]) and high SNR (*Δ* = 1.01° [<-1000, >1000]) target stimuli. The positive skew to the previous response was significant at all levels of SNR in the target stimulus. This was smallest for high SNR target stimuli (*ΔLow* = 0.91° [0.45, 1.44]; *ΔMedium* = 0.53° [0.11, 0.92]), and did not differ significantly between low and medium SNR target stimuli (*Δ* = 0.38° [-0.11, 01.01]).

There was no serial dependence to the inducer stimulus following a low confidence response and a marginal effect following a high confidence response, but these effects were not different to one another (*Δ* = 0.56° [-1.52, >1000]). The bias to the inducer response was significant for both levels of inducer confidence and these did not differ from one another (*Δ* = 0.42° [-0.07, 1.06]). There was a significant positive bias to the previous stimulus on low confidence trials and a small but positive effect on high confidence trials (0.15° [0.12, 0.84], and these two amplitudes did not differ (*Δ* = 0.49° [-0.03, 0.89]). There were significant biases to the previous response on both low and high confidence trials, and the effect was larger on low confidence trials (*Δ* = 1.05° [0.18, 1.83]).

**Supplementary Analysis 4**

In the main text, we examined the effects of subjective confidence by sorting confidence responses into the categories of “Low” and “High” based on whether they were smaller or greater than the participant’s mean confidence response. Here, we report the effects of confidence split by the actual confidence response made (1-4).

We first looked at how serial dependence differed based on the confidence response made on the inducer trial (Table S5). When looking at the skew towards previous stimuli, the derivative of von Mises model yielded no serial dependence following higher confidence responses (‘2’, ‘3’, or ‘4’) and a significant repulsive effect away from the inducer stimulus when confidence in the response was low (‘1’). This latter effect was significantly different to all other amplitudes (*Δ’2’* = 1.73° [0.10, 2.78]; *Δ’3’* = 2.40° [0.87, 3.71]; *Δ’4’* = 2.53° [0.41, 3.86]). As they were instructed to use the ‘1’ response only when they were guessing, we reasoned that this reflects a bias *towards* the previous trial’s adjustment response at all levels of subjective confidence, but the response deviates further from the actual stimulus shown when participants are responding randomly (confidence = ‘1’) than when they are seeing and responding to the stimulus (confidence > ‘1’). In line with this, when looking at the skew to previous responses there was positive serial dependence at all higher levels of inducer response confidence (‘2’, ‘3’, and ‘4’). The effect was significantly larger following ‘2’ responses than ‘4’ responses (*Δ* = 0.99° [0.17, 1.72]), but no other effects differed significantly (*Δ’2’-‘3’* = 0.57° [-0.16, 1.38]; *Δ’3’-‘4’* = 0.42° [-0.46, 1.23]). The effect was non-significant for ‘1’ responses despite being numerically similar, perhaps due to the relatively small number of ‘1’ responses made, and this did not differ from the effect following other confidence responses (*Δ*’2’ = 0.49° [-1.13, 1.77]; *Δ*’3’ = 0.08° [-1.42, 1.87]; *Δ*’4’ = 0.50° [-1.02, 2.27]). The model-free analysis yielded no significant biases to inducer stimuli and a significant positive bias to the previous response following ‘2’ (*t*[32] = 3.58, *p* < .001, *BF*_10_ = 58.09), ‘3’ (*t*[32] = 3.52, *p* < .001, *BF*_10_ = 50.41), and ‘4’ responses (*t*[32] = 2.18, *p* = .018, *BF*_10_ = 2.89), but not ‘1’ responses (*t*[32] = 0.90, *p* = .187, *BF*_10_ = 0.44). However, there was no difference between any of these effects (*F*[1.58,50.47] = 0.65, *p* = .490, *BF*_10_ = 0.25 [Greenhouse-Geisser Corrected]).

**Table S5**

*Serial Dependence Effects to the Inducer Stimulus and Response Based on Self-Reported Confidence on the Inducer Trial*

|  | Derivative of von Mises | | Model-Free |
| --- | --- | --- | --- |
| Confidence | Amplitude (°) [95% CI] | Bias (°) [95% CI] | |
| Stimulus-Based Analysis | | |  |
| ‘1’ | -2.01 [-3.21, -0.25] | | -0.39° [-1.79, 1.02] |
| ‘2’ | -0.28 [-1.37, 0.70] | | -1.67° [-3.70, 0.36] |
| ‘3’ | 0.49 [-0.35, 1.26] | | 0.40° [-0.29, 1.09] |
| ‘4’ | 0.52 [-0.54, 1.12] | | 0.82° [-0.50, 2.14] |
| Response-Based Analysis | | |  |
| ‘1’ | 1.22 [-0.22, 3.04] | | 1.08 [-1.36, 3.52] |
| ‘2’ | 1.71 [1.15, 2.45] | | 2.16 [0.93, 3.39] |
| ‘3’ | 1.14 [0.50, 1.87] | | 1.02 [0.43, 1.61] |
| ‘4’ | 0.72 [0.14, 1.42] | | 1.35 [0.09, 2.61] |

Turning to the effects of confidence on the target trial (Table S6), the derivative of von Mises model yielded no significant effects to the previous stimulus at any level, but positive biases to the previous response for all confidence response other than the lowest. None of these amplitudes differed from one another (*Δ’1’-‘2’* = 1.65° [-1.31, 4.78]; *Δ’1’-‘3’* = 1.69° [-1.26, 4.84]; *Δ’1’-‘4’* = 2.14° [-0.95, 5.34]; *Δ’2’-‘3’* = 0.04° [-0.69, 0.90]; *Δ’2’-‘4’* = 0.49° [-0.37, 1.49]; *Δ’3’-‘4’* = 0.45° [-0.28, 1.09]). In the model-free analysis there were no significant biases to the previous stimulus at any level of confidence on the target trial. There were significant positive biases to the previous response on trials with ‘3’ (*t*[32] = 3.57, *p* < .001, *BF*_10_ = 56.92) or ‘4’ (*t*[32] = 2.03, *p* = .026, *BF*_10_ = 2.20) confidence responses, but not ‘2’ (*t*[32] = 0.34, *p* = .630, *BF*_10_ = 0.15) or ‘1’ (*t*[32] = 0.73, *p* = .235, *BF*_10_ = 0.36) responses. However, these effects did not differ from one another (*F*[1.38,44.25] = 0.41, *p* = .593, *BF*_10_ = 0.08 [Greenhouse-Geisser Corrected]).

**Table S6**

*Serial Dependence Effects to the Inducer Stimulus and Response Based on Self-Reported Confidence on the Target Trial*

|  | Derivative of von Mises | | Model-Free |
| --- | --- | --- | --- |
| Confidence | Amplitude (°) [95% CI] | Bias (°) [95% CI] | |
| Stimulus-Based Analysis | | |  |
| ‘1’ | 2.74 [-1.19, 6.38] | | -0.44 [-4.79, 3.91] |
| ‘2’ | 0.43 [-0.79, 1.58] | | -0.01 [-1.08, 1.07] |
| ‘3’ | 0.40 [-0.45, 1.03] | | 0.39 [-0.20, 0.97] |
| ‘4’ | -0.11 [-0.93, 0.56] | | -0.39 [-1.58, 0.81] |
| Response-Based Analysis | | |  |
| ‘1’ | 3.00 [-0.05, 6.27] | | 1.04 [-1.86, 3.94] |
| ‘2’ | 1.35 [0.74, 2.27] | | -0.73 [-5.15, 3.70] |
| ‘3’ | 1.31 [0.74, 1.96] | | 1.29 [0.56, 2.03] |
| ‘4’ | 0.86 [0.41, 1.51] | | 0.80 [-0.00, 1.60] |

As in the main text, we find no convincing evidence that confidence on the inducer trial affected serial dependence. However, we also find no differences based on the confidence in the target trial, which likely differs from the main text due to the relatively small trial numbers included in each condition here.

**Supplementary Analysis 5**

We conducted the same analyses as in sections 3.2-3.4 of the main text on the data including all valid trials (i.e., trials with super-fast response times were still excluded, as was the first trial of each block, but no trials were excluded based on the size of the error made). This data is shown in Tables S7-S10. There were some slight differences in the findings yielded by the derivative of von Mises model. In the main analysis, serial dependence had been significantly larger on trials with low positive evidence targets; here, the difference between positive evidence conditions remained numerically large but did not reach significance. The effect of SNR in the target was actually stronger in the full data than in the main analyses, as the stimulus-based analysis yielded three significantly different amplitudes. Finally, in the stimulus-based analysis only, the effect of subjective confidence on the target trial was no longer significant. The stimulus-based analyses using the model-free method did not differ from the main text. The response-based analyses using the model-free method changed slightly, as the effects of SNR and subject confidence in the target stimulus were no longer significant.

**Table S7**

*Effects of Positive Evidence, SNR, and Subjective Confidence in the Inducer and Target Stimulus on Serial Dependence to Stimuli Using the Derivative of von Mises Model*

|  |  |  | Amplitude | Differences |
| --- | --- | --- | --- | --- |
|  |  |  | (°) [95% CI] | |
| Positive Evidence | Inducer | Low | 0.19 [-0.95, 1.46] |  |
|  |  | High | 0.15 [-1.31, 1.43] |  |
|  | Target | Low | 0.85 [-0.10, 1.84] |  |
|  |  | High | -0.49 [-1.62, 0.61] |  |
| SNR | Inducer | Low | 0.11 [-0.88, 1.12] |  |
|  |  | Medium | 0.17 [-0.94, 1.26] |  |
|  |  | High | 0.51 [-0.35, 1.41] |  |
|  | Target | Low | 1.63 [0.24, 3.07] | Low-Med = 1.45 [0.26, 2.68] |
|  |  | Medium | 0.18 [0.72, 1.03] | Low-High = 2.62 [1.50, 3.68] |
|  |  | High | -0.99 [-1.94, -0.01] | Med-High = 1.17 [0.53, 1.80] |
| Confidence | Inducer | Low | 0.11 [-1.01, 1.27] |  |
|  |  | High | 0.42 [-0.64, 1.36] |  |
|  | Target | Low | 0.99 [-0.23, 2.16] |  |
|  |  | High | -0.26 [-1.19, 0.54] |  |

**Table S8**

*Effects of Positive Evidence, SNR, and Subjective Confidence in the Inducer and Target Stimulus on Serial Dependence to Responses Using the Derivative of von Mises Model*

|  |  |  | Amplitude | Differences |
| --- | --- | --- | --- | --- |
|  |  |  | (°) [95% CI] | |
| Positive Evidence | Inducer | Low | 1.07 [0.41, 1.87] | Low-Med = 0.01 [-0.91, 0.91] |
|  |  | High | 1.06 [0.30, 1.98] |  |
|  | Target | Low | 1.57 [0.79, 2.63] | Low-Med = 0.75 [-0.09, 1.76] |
|  |  | High | 0.82 [0.18, 1.59] |  |
| SNR | Inducer | Low | 1.11 [0.44, 1.88] | Low-Med = 0.05 [-0.62, 0.79] |
|  |  | Medium | 1.06 [0.48, 1.75] | Low-High = -0.29 [-1.06, 0.51] |
|  |  | High | 1.40 [0.65, 2.30] | Med-High = -0.34 [-1.00, 0.24] |
|  | Target | Low | 1.83 [0.90, 2.86] | Low-Med = 0.63 [-0.33, 1.69] |
|  |  | Medium | 1.17 [0.58, 1.94] | Low-High = 1.29 [0.33, 2.32] |
|  |  | High | 0.54 [-0.14, 1.30] | Med-High = 0.63 [0.02, 1.20] |
| Confidence | Inducer | Low | 1.53 [0.90, 2.19] | Low-High = 0.60 [-0.14, 1.20] |
|  |  | High | 0.93 [0.35, 1.79] |  |
|  | Target | Low | 1.75 [0.78, 2.81] | Low-High = 1.02 [0.01, 1.97] |
|  |  | High | 0.73 [0.32, 1.35] |  |

**Table S9**

*Effects of Positive Evidence, SNR, and Subjective Confidence in the Inducer and Target Stimulus on Serial Dependence to Stimuli Using the Model-Free Method*

|  |  |  | Bias (°) [95% CI], *p*-val, BF_10_ | | Difference |
| --- | --- | --- | --- | --- | --- |
| Positive Evidence | Inducer | Low | 0.24 [-0.48, 0.24], .249, 0.34 | | |
|  |  | High | 0.00 [-0.48, 0.89], .269, 0.32 | | |
|  | Target | Low | 0.48 [-0.17, 1.14], .070, 0.96 | | |
|  |  | High | 0.10 [-0.54, 0.74], .378, 0.31 | | |
| SNR | Inducer | Low | 0.22 [-0.38, 0.82], .229, 0.37 | | No main effect of SNR (*p* = .171) |
|  |  | Medium | 0.23 [-0.44, 0.90], .247, 0.35 | |  |
|  |  | High | 0.75 [0.19, 1.31], .005, 8.27 | |  |
|  | Target | Low | 0.87 [0.04, 1.70], .020, 2.64 | | Low-Med: *p* = .647 |
|  |  | Medium | 0.39 [-0.22, 1.00], .098, 0.73 | | Low-High: *p* = .010 |
|  |  | High | -0.21 [-0.80, 0.37], .767, 0.12 | | Med-High: *p* = .134 |
| Confidence | Inducer | Low | 0.05 [-0.51, 0.62], .424, 0.22 |  | |
|  |  | High | 0.52 [-0.27, 1.30], .094, 0.76 | | |
|  | Target | Low | 0.69 [0.08, 1.30], .286, 0.31 | | *p* = .065 |
|  |  | High | 0.16 [-0.41, 0.73], .014, 3.70 | | |

**Table S10**

*Effects of Positive Evidence, SNR, and Subjective Confidence in the Inducer and Target Stimulus on Serial Dependence to Responses Using the Model-Free Method*

|  |  |  | Bias (°) [95% CI], *p*-val, BF_10_ | Difference |
| --- | --- | --- | --- | --- |
| Positive Evidence | Inducer | Low | 1.26 [0.68, 1.84], <.001, >150 | *p* = .282 |
|  |  | High | 0.97 [0.38, 1.56], .001, 34.08 | |
|  | Target | Low | 1.55 [0.90, 2.20], <.001, >150 | *p* = .062 |
|  |  | High | 0.93 [0.52, 1.34], <.001, >150 | |
| SNR | Inducer | Low | 1.22 [0.72, 1.73], <.001, >150 | No main effect of SNR (*p* = .924) |
|  |  | Medium | 1.11 [0.57, 1.66], <.001, >150 |  |
|  |  | High | 1.21 [0.65, 1.77], <.001, >150 |  |
|  | Target | Low | 1.40 [0.75, 2.06], <.001, >150 | No main effect of SNR (*p* = .062) |
|  |  | Medium | 1.22 [0.76, 1.69], <.001, >150 |  |
|  |  | High | 0.75 [0.24, 1.26], .002, 15.88 |  |
| Confidence | Inducer | Low | 1.28 [0.86, 1.70], <.001, >150 | *p* = .293 |
|  |  | High | 1.06 [0.59, 1.53], <.001, >150 | |
|  | Target | Low | 1.31 [0.78, 1.84], <.001, >150 | *p* = .150 |
|  |  | High | 0.99 [0.59, 1.39], <.001, >150 | |
